# Patterns of inflammation and immune activation by coreceptor use in people living with HIV-1

**DOI:** 10.3389/fimmu.2025.1632287

**Published:** 2025-07-10

**Authors:** Francisco Xavier Guerra-Castillo, Sandra Pinto-Cardoso, Santiago Ávila-Ríos, Monserrat Chávez-Torres, Amy Peralta-Prado, Carolina González-Torres, Javier Gaytán-Cervantes, Brenda Requena-Benitez, Dafne Díaz-Rivera, Carmen Alaez-Verson, María Concepción Hernández-García, Vilma Carolina Bekker-Méndez

**Affiliations:** ^1^ Unidad de Investigación Médica en Inmunología e Infectología, Hospital de Infectología “Dr. Daniel Méndez Hernández”, Centro Médico Nacional La Raza, Instituto Mexicano del Seguro Social (IMSS), Ciudad de México, Mexico; ^2^ Posgrado en Ciencias Biológicas, Unidad de Posgrado, Universidad Nacional Autónoma de México (UNAM), Ciudad de México, Mexico; ^3^ Centro de Investigación en Enfermedades Infecciosas, Instituto Nacional de Enfermedades Respiratorias Ismael Cosío Villegas, Ciudad de México, Mexico; ^4^ Laboratorio de Secuenciación, División de Desarrollo de la Investigación en Salud, Centro Médico Nacional Siglo XXI, Instituto Mexicano del Seguro Social, Ciudad de México, Mexico; ^5^ Laboratorio de Diagnóstico Genómico, Instituto Nacional de Médicina Genómica (INMEGEN), Ciudad de México, Mexico; ^6^ Hospital de Infectología “Dr. Daniel Méndez Hernández”, Centro Médico Nacional La Raza, Instituto Mexicano del Seguro Social (IMSS), Ciudad de México, Mexico

**Keywords:** HIV-1 tropism, CXCR4, CCR5, chronic immune activation, IL-6, CD38, HLA-DR, next generation sequencing

## Abstract

**Introduction:**

Human immunodeficiency virus type 1 (HIV-1) utilizes either the CCR5 (R5) or CXCR4 (X4) coreceptor for host cell entry. Coreceptor switching from R5 to X4 and elevated immune activation have been associated with disease progression. X4-tropic HIV-1 is predominantly observed in the late stage of infection, when the immune environment characterized by chronic activation is optimal for their replication. The aim of this study was to determine viral tropism in late HIV presenters and who have not previously received treatment in Mexico City and its relationship with markers of chronic immune activation.

**Methods:**

A cross-sectional study was conducted on 122 people living with HIV (PLWH) recruited from two public health services. Viral tropism was determined using next-generation sequencing (NGS) and the geno2pheno algorithm. Immune activation was assessed through flow cytometry (CD38+, HLA-DR+), and soluble markers (sCD14, sCD163, IL-6) were quantified using enzyme-linked immunosorbent assays (ELISA). Differences in immune activation patterns between R5 and X4 group were explored using Mann-Whitney Wilcoxon test and t-test, and a principal component analysis (PCA). Logistic regression was used to evaluate associations between immune activation profiles and the presence of X4-tropic viruses.

**Results:**

Ninety-eight individuals had high-quality V3 loop sequences, 81.6% harbored only R5 variants (R5 group), while 18.4% had mixed R5/X4 populations (X4 group). Most PLWH had CD4+ T cell counts below 200 cells/µL, showing no significant difference between groups. Elevated levels of IL-6 were significantly associated with the R5 group (p = 0.01), while the X4 group showed increased expression of CD38+ and HLA-DR+CD38+ markers, although not statistically significant. Furthermore, IL-6 emerges as a negative predictor for the presence of X4 viruses (OR = 0.06, p = 0.006).

**Conclusion:**

R5-tropic viruses are associated with elevated inflammatory responses in early stages, as indicated by higher IL-6 levels, while X4-tropic viruses may contribute to CD4+ T cell depletion through immune activation. Consequently, elevated levels of IL-6 emerge as a negative predictor for the presence of X4 viruses. The relationship between viral tropism and chronic immune activation in HIV-1 infection reflects a complex interplay which appears to be bidirectional.

## 1 Introduction

In 2023, The Joint United Nations Programme on human immunodeficiency virus (HIV) and acquired immunodeficiency syndrome (AIDS) reported the estimated prevalence of 380,000 of people living with HIV (PLWH) across all ages in Mexico (1). Of those, 64% of PLWH are on antiretroviral therapy (ART) and 59% have suppressed viremia (defined as plasma viral load below 40 copies of HIV-1 RNA/mL) (1). The human immunodeficiency virus type 1 (HIV-1) requires both the CD4 receptor and a co-receptor, either C-C chemokine receptor 5 (CCR5) or C-X-C chemokine receptor 4 (CXCR4) to infect hosts cells. The preference for either co-receptor is referred to as viral tropism. HIV viral tropism is underreported in Mexico. This is of concern as it is crucial for understanding HIV pathogenesis and disease progression, particularly in a country where individuals are diagnosed with advanced HIV disease (late presenters) (1). The entry is mediated by the glycoproteins (gp) gp120-gp41 (2). HIV-1 transmitted/founder (T/F) virus typically uses CCR5 receptor (R5 virus) for entry (3), which dominates during the first weeks or years of HIV-1 infection. The virus can evolve and switch to use CXCR4 receptor (X4 virus) if the X4 virus has a higher fitness. The X4 phenotype is mostly seen in late stage of HIV infection (4, 5). Elevated immune activation and coreceptor switch have been associated with HIV-1 disease progression (6–10). There are three hypotheses that attempt to explain the processes underlying coreceptor switch and its association with disease progression, based on empirical evidence or model support. First, molecular determinants located in the V3 loop of gp120 confer the tropism coreceptor phenotype and few genetic changes in the sequence are required to determine which coreceptor is used to trigger the process of entry (2, 11). These changes are assumed to be the result of evolution of the viral populations during infection, where the quick turnover and high mutation rate of the virus increase the probability of X4 virus emergence as a random event during any stage of the disease (12, 13). Second, in immunocompetent people living with HIV (PLWH), X4 viruses do not have the fitter phenotype and are better recognized by the immune system than R5 viruses (7, 14). The CD8+ T cell response against X4 virus replication appears to be more effective than that against R5 virus. However, the progressive erosion of the immune system leads to reduced susceptibility to X4 virus (7, 15). If the X4 virus exhibits higher replicative fitness than the R5 counterparts, it could lead to its emergence and, in some cases, replace the predominance of the R5 virus variants (13, 16, 17). Third, HIV-1 exhibits different preferences for target cells, whereby CD4+ T-cells represent the primary target. Naive CD4+ T-cells express high levels of CXCR4+, while memory CD4+ T cells express both CXCR4+ and CCR5+ (18). Over time, the activation of naive CD4+ T cells into memory CD4+ T cells increases, as does the frequency of their proliferation. This leads to selective pressure in favor of the R5 virus, as the number of target cells increases. In the later stages of the infection, the proliferation rate of naive CD4+ T cells further increases, shifting the selection pressure in favor of X4 variants (19–22). This switch from CCR5 to CXCR4 coreceptor is associated with rapid CD4+ T cell depletion and disease progression. Chronic immune activation characterizes HIV-1 disease progression and is often measured by the expression of the surface markers HLA-DR and CD38 on both CD4+ and CD8+ T cells (22). In addition to the aforementioned surface markers, other surface markers have been identified as indicators of T cell exhaustion (programmed death-1, PD-1) and senescence (CD57+). The expression of these markers has been implicated in the impairment of effector functions and T cell terminal differentiation and extensive proliferation, thereby contributing to the compromise of the immune system’s ability to control viral replication (22–26). Furthermore, a broad spectrum of biomarkers have been used to quantify chronic immune activation, and these biomarkers remain elevated even in PLWH with antiretroviral therapy (ART) with undetectable viraemia (27). Among these biomarkers, interleukin-6 (IL-6) is of significant interest. IL-6 is a cytokine that plays a critical role in activating and regulating the immune response to viral infections. Numerous studies have demonstrated a correlation between IL-6 and mortality and/or morbidity in PLWH (28, 29). In addition, microbial translocation (the passage of bacteria and/or microbial products from the gut into the systemic circulation) triggers the activation of monocytes and/or macrophages during the early phase of the disease, resulting in the shedding of sCD14 and sCD163 (22). sCD14 is a marker of monocyte activation, is shed in response to lipopolysaccharide (LPS) stimulation which reflect CD4+ T cell depletion in the gut-associated mucosal lymphoid tissue (GALT) and correlates with the expression of HLA-DR and CD38 on CD8+ T cells (27, 30). Soluble CD163 (sCD163) is a marker of HIV activity reflecting the virus replication and T cell activation (31). Several studies have reported discrepancies regarding whether X4 variants are the cause or consequence of a dysfunctional immune response. Recent studies have examined the relationship between the presence of X4 variants and disease outcome. In 2017, Hayashida, et al. (32) reported that the rate of decrease of CD4+ T cell count or the rise of HIV-1 load accelerated significantly after the emergence of X4 variants in two individuals experiencing slow progression. Also, in 2020, Connell et al. (7) demonstrated that X4-tropism is not the favorable viral phenotype in the immunocompetent host. They observed that higher levels of immune activation can predict the subsequent selection of X4 variants. Therefore, it is essential to have a better understanding of the mechanisms that drive viral infection via X4 variants, from minority to dominant viral populations. Evidence suggest that X4 variants exhibit a low replication rate in early HIV-1 infection. This is due to the fact that X4 variants target preferentially naive CD4+ T cells, which results in insignificant viral production (33). For this reason, next generation sequencing (NGS) platforms are well-suited for the characterization and genotyping of viral diversity. Furthermore, bioinformatic algorithms such as geno2pheno (34) are used to predict the tropism in the viral population. Consequently, X4 variants can be detected at very low levels shortly after HIV transmission. Also, X4 variants have been observed to exhibit increased pathogenicity which contributes to CD4+ T cell depletion. This, in turn, leads to the proliferation of target cells that are adequate for replication in terms of R5 and X4 variants. Such changes may create an environment in the host that maintains chronic immune activation, which creates the optimal conditions for switching coreceptor preference. Immune activation is critical for HIV-1 to maintain replication and cellular infection. To determine whether early immune activation is associated with the presence of X4 variants, it is crucial to examine the most important cellular markers, in particular HLA-DR and CD38, in CD4+ and CD8+ T cells. Additionally, the significance of soluble markers such as sCD163, sCD14 and IL-6, which are associated with inflammation and the activation of antigen-presenting cells in PLWH with the presence of X4 variants, has yet to be fully elucidated. The aim of this study is to determine the levels of immune activation and their relationship with viral tropism in PLWH who have not been previously treated in México City. We used NGS to determine the viral tropism of HIV; enzyme-linked immunosorbent assays to quantify soluble markers of inflammation and immune activation; and flow cytometry to quantify T cell immune activation, senescence, exhaustion, and expression of coreceptors (CCR5+ and CXCR4+).

## 2 Materials and methods

### 2.1 Study participants

One hundred and twenty-two people living with human immunodeficiency virus (PLWH) were enrolled from two public health services from the Metropolitan area of Mexico City, the Instituto Mexicano del Seguro Social (IMSS) and the Instituto Nacional de Enfermedades Respiratorias (INER). These were PLWH who had recently been diagnosed with HIV-1 (within the last 12 months) and most were late presenters. This is concordant with the epidemiology of HIV in Mexico, where most PWLH are diagnosed in advanced HIV disease (1). Both cohorts were recruited between 2016 and 2019 in accordance with Good Clinical Practices. All subjects provided Informed Consent to participate, and the study was reviewed and approved by the Institutional Ethics Committees of IMSS (registration number, CNIC-R-785-094) and INER (C59-15). Clinical and demographic data were obtained from medical records. All PLWH were naive to antiretroviral therapy and had no prior exposure to ART. No explicit exclusion criteria were defined for this study.

### 2.2 Samples

Six milliliters of peripheral blood were collected by venipuncture in two ethylenediaminetetraacetic acid (EDTA) tubes. Blood samples were processed immediately to separate the plasma by centrifugation at 3,500 rpm for 10 min at room temperature which was immediately preserved at -80°C. Peripheral blood mononuclear cells (PBMC) were isolated by Ficoll density gradient centrifugation (STEMCELL Technologies, Vancouver, Canada) according to manufacturer’s protocol, and cryopreserved at -196°C.

### 2.3 Library preparation

Frozen plasma samples were thawed at room temperature. Samples were centrifuged at 14,000 rpm at 4 °C for 2 hours. HIV-1 RNA was extracted using QIAamp viral RNA minikit (Qiagen, Valencia, CA) according to the manufacturer´s instructions. Primers and PCR conditions were previously described and listed elsewhere (35). A one-step reverse-transcriptase-polymerase chain reaction was performed to amplify a product of 1692 bp using the one-step Superscript III Reverse Transcriptase (Invitrogen, Carlsbad, CA, USA) as a first round. Then, a nested PCR was performed to amplify the region encoding the HIV-1 V3 loop (HXB2, Env gene, GenBank: K03455.1, 7062:7373) using a High-fidelity PCR polymerase (Roche Diagnostics, Mannheim, Germany). PCR products of 379 base pairs corresponding to the PCR amplicon of the V3 loop (overhang adapter sequences were added to the locus-specific primer) were visualized by electrophoresis on a 1.5% agarose gel. PCR products were purified using an AgenCourt AMPure XP PCR purification beads (Beckman Coulter, Brea, CA) using a size selection protocol as instructed in the NEB Next Ultra DNA Library Prep Kit (Illumina, E7370). PCR products were quantified with Qubit ds DNA Assay Kit (ThermoFisher, Waltham, MA, USA) and used for one round of PCR amplification with Nextera XT Index Kit (Illumina, Santa Clara, CA, USA) and Kapa High-Fidelity PCR Kit (Kapa Biosystems, Woburn, MA) according to manufacturer’s protocol. Libraries were purified using AgenCourt AMPure XP PCR purification beads (Beckman Coulter, Brea, CA) and library size was verified using the 4200 TapeStation (Agilent, Santa Clara, CA, USA). Libraries were normalized at a final concentration of 4 nM and paired-end sequencing was performed with the MiSeq Reagent Kits v.2 (500 cycles) (Illumina, Santa Clara, CA, USA) with 30% of PhiX as a control.

### 2.4 Sequence data analysis and coreceptor prediction usage

Raw FASTQ files were processed using FastQC (version 0.11.5) for quality control checks. Next, sequences were filtered, adapters removed using Trimmomatic (version 0.39) and merged using BBTools (BBMap, version 38.87). Geno2pheno was used to predict coreceptor use (https://coreceptor.geno2pheno.org) (34, 36). Coreceptor usage was reported using the next generation sequencing data recommendations reported elsewhere (37–39). We used a False Positive Rate (FPR) cut-off of ≤3.75% for prediction of X4 variants and a cut-off of >2% for the viral population (37, 38).

### 2.5 Enzyme-linked immunosorbent assay

Several markers were measured in plasma samples: sCD163 (Human sCD163 Quantikine ELISA Kit, R&D Systems; Minneapolis, Minnesota, USA), sCD14 (Hu sCD14 Quantikine ELISA R&D Systems; Minneapolis, Minnesota, USA) and IL-6 (IL-6 Human ELISA kit High Sensitivity, ThermoFisher) according to manufacturer’s instructions. Plasma samples were diluted 250-fold for sCD14, 25-fold for sCD163 and 2-fold for IL-6. Each assay was performed in duplicate. Assays were read at 450nm (Epoch, BioTek, Winooski, Vermont, USA) and analyzed using the Gen5 software, version 2.07. Wavelength correction was applied by subtracting the optical density at 570nm.

### 2.6 Flow cytometry

A 14-color panel including 13 monoclonal antibodies (BD Biosciences): CD3, CD4, CD8, CD69, CD25, HLA-DR, CD38, CD45RO, CD197 (CCR7), PD-1, CD57, CCR5 (CD195), CXCR4 (CD184) and a Live/Dead stain kit was used. Frozen PBMCs were thawed and rested for at least 30 min. Cells were washed in 15 mL of phosphate-buffered solution (PBS), centrifuged at 500 *x g* for 5 min at room temperature. After discarding the supernatant, cells were stained with a cocktail of monoclonal extracellular antibodies for 20 min in the dark. Cells were washed twice in PBS, and fixed in 300 µL of 1% paraformaldehyde (Sigma-Aldrich, St. Louis, Missouri, USA) and kept in the dark at 4°C until acquisition. Flow cytometry acquisition was performed on a BD FACS Aria Fusion (BD Biosciences, San Jose, CA, USA) within 24 h of staining. A minimum of 3 million events were acquired per sample. Quality controls were performed using BD Cytometer Setup and Tracking Beads and Rainbow Beads (BD Biosciences). A compensation matrix was calculated and applied using BD Comp Beads (BD Biosciences). Data was analyzed using FlowJo™ v10.9 (BD, Ashland, Oregon, United States). Raw FCS (flow cytometry standard) files were first quality-controlled using FlowAI v2.3.1 with default parameters. Gating strategy is described in **Supplementary Figure 1**.

### 2.7 Statistical data analyses

Continuous data were tested for normal distribution. Variables with skewed data were log-transformed. Missing data were handled by imputation using K-nearest neighbor imputation (VIM, version 6.2.2) (40, 41). Mann-Whitney Wilcoxon test and t-Test univariate analysis were performed for continuous data. Spearman or Pearson correlation tests were performed to evaluate the relationship between two continuous variables, the p-values were adjusted with the Benjamini-Hochberg or false discovery rate (FDR) method, as applicable (cor.test, wilcox.test and t.test [stats], version 4.3.2). Graphical display of correlation matrix was created using corrplot v0.92 in R. Principal component analysis (PCA) was performed (prcomp [stats], version 4.3.2; fviz_eig [factoextra], version 1.0.7) and tested with PCAtest (version 0.0.1), which is an R package that implements permutation-based statistical tests to evaluate the overall significance of a PCA (42). Finally, logistic regression was performed using the X4 tropism as a dichotomous dependent variable, defined by the cut-off of ≤3.75% and the immunological and clinical data as independent variables, and adjusted for age (lm [stats], version 4.3.2). We did not adjust for sex, as all the individuals were men. A statistically significant difference was defined as a p value of <0.05. We performed all statistical test with the stats package, and the visualization and graphics were conducted with ggplot2 (version 3.4.4).

## 3 Results

### 3.1 Demographic and clinical characteristics of the PLWH

Demographic and clinical characteristics are summarized in **Table 1**. We included 122 PLWH, all of whom were male with a median age of 30 years. HIV-1 acquisition risk was predominantly among men who have sex with men (MSM, 84.4%). The median CD4+ T cell count was 89 cells/µL, with 68% of PLWH having CD4+ T cell counts below 200 cells/µL. The median plasma viral load (pVL) was 5.29 log RNA copies/mL.

**Table 1:** Demographics and Clinical characteristics of PLWH with no prior exposure to antiretrovirals.

	All individuals	IMSS	INER	p value
No. of individuals	122	57	65	
Sex assigned at birth: male n (%)	122 (100)	57(100)	65(100)	
Age median (IQR)	30 (25 - 38)	28 (23 - 34)	34 (28 - 42.5)	<0.001
HIV-1 Acquisition Risk n(%)				
MSM	103 (84.42)	46 (80.7)	57 (87.7)	
Heterosexual	13 (10.65)	7 (12.3)	6 (9.2)	
Bisexual	4 (3.27)	4 (7)	0	
NA	2 (1.64)	0	2 (3.1)	
CD4+ count (cells/µL) median (IQR)	89 (42 - 235)	152 (77 - 361)	65 (34.75-131.75)	<0.001
CD4 <200 n(%)	83 (68.03)	32 (56.14)	51 (78.50)	
CD4 200 - <500 n(%)	28 (22.95)	19 (33.33)	9 (13.80)	
CD4 ≥500 n(%)	6 (4.91)	6 (10.52)	0 (0)	
CD4 NA n(%)	5 (4.09)	0 (0)	5 (7.70)	
CD8+ count (cells/µL) median (IQR)	761(445.5 – 1326.35)	970 (475-1434.2)	694 (468-1184.5)	0.168
CD4/CD8 ratio median (IQR)	0.13 (0.07-0.26)	0.22 (0.09-0.38)	0.105 (0.05-0.16)	<0.001
pVL (copies/mL) median (IQR)	195210 (68471.5-575240.8)	148090 (35138-277404)	227989 (120115.5-676495.5)	0.011
pVL log (copies/mL) median (IQR)	5.29 (4.83 - 5.76)	5.17 (4.54-5.44)	5.36 (5.08-5.83)	0.011

NGS, next generation sequencing; FPR, false positive rate; CCR5, C-C chemokine receptor 5; CXCR4, C-X-C chemokine receptor 4; IQR, Interquartile range; pVL, plasma viral load. CD, cluster of differentiation; NA, not available; MSM, men who have sex with men. Comparisons were made using Mann-Whitney U test or t-test as appropriate and using significant p-values <0.05.

The 122 individuals came from two hospitals which care for PLWH, IMSS (n = 57) and from INER (n = 65) (**Table 1**). There were significant differences between the two groups. The median age of IMSS individuals was 30 years, whereas the median of INER individuals was age 34 years (interquartile range (IQR), 23–34 versus 28-42.5 years; p < 0.001). The INER group exhibited a lower immune status, as assessed by CD4+ T cell count, with a median of 65 cells/µL compared to 152 cells/µL (IQR, 77–361 versus 34.75-131.75; p = <0.001). Additionally, the proportion of individuals in the INER group with a CD4+ T cell count below 200 cells/µL was 78.5%, compared to 56.1% in the IMSS group. None of the individuals in the INER group had a CD4+ T cell count above 500 cells/µL. The viral load was found to be higher in the INER group, with a median of 5.36 log copies/mL, in comparison to 5.17 log copies/mL in the IMSS group (IQR, 4.54-5.44 vs 5.08-5.83; p = 0.011).

### 3.2 Distribution and frequency of R5 and X4 viral variants by sequencing

**Figure 1:**
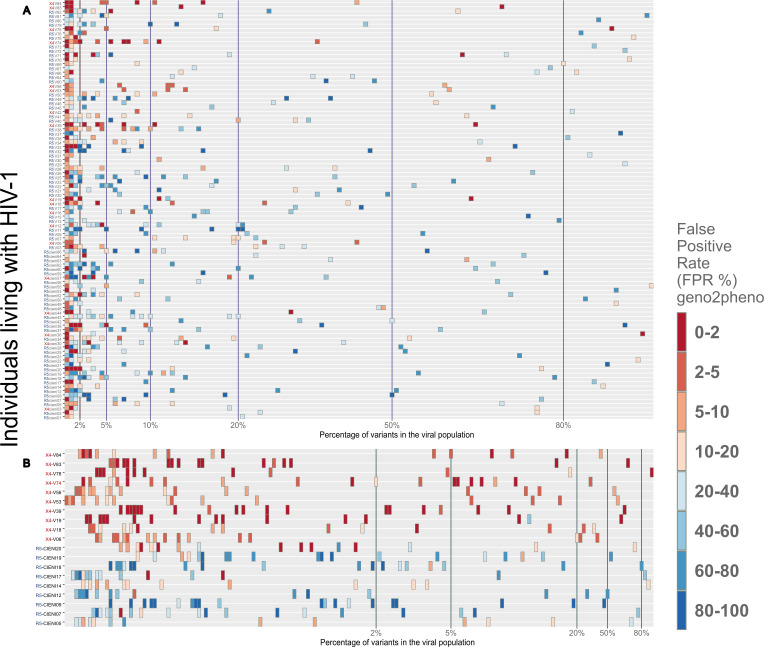
Distribution of R5 and X4 viral variants among the viral population for each individual. All samples were obtained from late HIV presenters. **(A)** The 20th most frequent viral variants from each individual were plotted, as the remaining variants had a frequency of less than 1% of the viral population (see **Supplementary Material**). In order to infer the use of CXCR4 or CCR5 coreceptor in each of these sequence populations, the geno2pheno tool was employed, with a FPR threshold of <3.75%. It was observed that the majority of the viral variants present in each sample clustered below 2% of the viral population. The minority variants, which clustered between 2% and 20%, represented the second most abundant viral variants. **(B)** A closer examination of 19 samples, of which 10 were stratified as X4 (red) and 9 as R5 (blue). This figure confirmed that the majority of viral variants had a frequency below 2% of viral population.

Sequences were generated from an amplicon of 312 bp, which extends across the V3 region of the gp120. The geno2pheno_[454]_ algorithm was used to obtain a FPR value for each sequence, which was then used to infer the R5 or X4 entry phenotype for the virus. The median number of high-quality reads for R5 and X4 was 91203.5 and 150212, respectively. Additionally, the X4 group exhibited a greater diversity of viral variants compared to the R5 group (2870 vs 1577, respectively; p = 0.022) (see **Supplementary Table 1**). A total of 98 samples with high-quality V3 loop sequences were selected, given the number of quality reads compared with the total number of reads, which was one of the results of the geno2pheno algorithm. We stratified 80 individuals (81.6%) and predicted that HIV-1 exclusively harbors R5 viral variants. The remaining 18 individuals (18.4%) had a mixed population of R5 and X4 viral variants (**Figure 1**). Viral population sequencing revealed that 63.3% (62/98) individuals exhibited a single dominant R5 viral variant, defined as a variant present in more than 50% of the viral population. However, in one individual, two dominant R5 viral variants were identified. In contrast, among the five (5.1%) individuals who showed a single dominant X4 viral variant, one individual exhibited two co-dominant variants, one X4 and one R5. The remaining 12 individuals from the X4 group (66.7%) exhibited a dominant R5 viral variant coexisting with X4 minority variants. Additionally, the minority variants of significance, defined as variants that fall between the 2-20% frequency cut-off, were the most prevalent in the viral population among most of the individuals. Furthermore, the frequency of R5 viral variants was significantly higher than that of X4 viral variants, with 214 variants versus 37, respectively. The remaining viral variants in the population consisted of hundreds of low-frequency viral forms, which lie below the 2% cut-off, and most of these were R5 viral variants (n = 1398) compared to X4 viral variants (n = 148) that we found in this particular analysis with the 20 most frequent viral variants.

### 3.3 Demographic, clinical characteristics and quantification of soluble markers of immune activation according to tropism stratification

Regarding clinical characteristics, the majority of PLWH in both groups exhibited CD4+ T cell counts below 200 cells/µL and elevated pVL, as shown in **Table 2**. No significant differences were observed between the R5 and X4 groups with respect to the rest of demographic and clinical characteristics. Soluble markers (**Table 3**), with the exception of sCD14 levels, exhibited elevated levels in the R5 group relative to the X4 group, particularly IL-6 (3.28 vs 2.42 pg/mL; p = 0.01).

**Table 2:** Demographics and Clinical characteristics of PLWH with no prior exposure to antiretrovirals.

	Individuals with NGS tropism results	CCR5 (FPR>3.75)	CXCR4 (FPR<3.75)	p value
**No. of individuals**	98	80	18	
Sex assigned at birth: male n (%)	98 (100)	80(100)	18 (100)	
Age median (IQR)	30 (25 - 37)	29.5 (25 -38)	33 (28.25 – 35.5)	0.627
HIV-1 Acquisition Risk n(%)				
MSM	79 (80.6)	66 (82.5)	13 (72.22)	
Heterosexual	13 (13.3)	10 (12.5)	3 (16.6)	
Bisexual	4 (4.1)	2 (2.5)	2 (11.11)	
NA	2 (2.0)	2 (2.5)	0 (0)	
CD4+ count (cells/µL) median (IQR)	97 (43.3-259.75)	97 (42.65 – 272)	100 (68 – 250)	0.977
CD4 <200 n (%)	62 (63.3)	50 (62.5)	12 (66.66)	0.762
CD4 200 - <500 n (%)	25 (25.5)	20 (25.0)	5 (27.77)	0.488
CD4 ≥500 n (%)	6 (6.1)	5 (6.25)	1 (5.55)	NA
CD4 NA n (%)	5 (5.1)	5 (6.25)	0 (0)	NA
CD8+ count (cells/µL) median (IQR)	766 (475-1333.6)	733.9 (420.2 – 1292.43)	970 (666.5 – 1512.35)	0.413
CD4/CD8 ratio median (IQR)	0.138 (0.080-0.297)	0.143 (0.08-0.278)	0.101(0.06-0.37)	0.697
pVL (copies/mL) median (IQR)	179997 (69630-473911.5)	202568.5 (64668.25 – 586108.5)	135893.5 (93716.75 – 268731.5)	0.406
pVL log (copies/mL) median (IQR)	5.26 (4.84-5.68)	5.3 (4.81 – 5.76)	5.13 (4.97 – 5.42)	0.406

NGS, next generation sequencing; FPR, false positive rate; CCR5, C-C chemokine receptor 5; CXCR4, C-X-C chemokine receptor 4; IQR, Interquartile range; pVL, plasma viral load. CD, cluster of differentiation; NA, not available; MSM, men who have sex with men. Comparisons were made using Mann-Whitney U test or t-test as appropriate and using significant p-values <0.05.

**Table 3:** Soluble markers of PLWH with no prior exposure to antiretrovirals.

	Individuals with NGS tropism results (n=98)	CCR5 (FPR>3.75) (n=80)	CXCR4 (FPR<3.75) (n=18)	p value
sCD163 (ng/mL) median (IQR)	1126.878 (820.46-1334.79)	1143.59 (909.62-1428.39)	936.47 (775.74-1263.77)	0.364
sCD14 (ng/mL) median (IQR)	1616.15 (1255.82-1852.36)	1616.15 (1322.61-1868.66)	1616.88 (1121.97-1800.47)	0.503
IL-6 (pg/mL) median (IQR)	3.28 (2.42-3.96)	3.28 (2.51-3.96)	2.42 (1.26-3.60)	**0.010**

IQR, Interquartile range; NGS, next generation sequencing; FPR, false positive rate; CCR5, C-C chemokine receptor 5; CXCR4, C-X-C chemokine receptor 4; IL, Interleukin; sCD, soluble cluster of differentiation. Comparisons were made using Mann-Whitney U test or t-test as appropriate and using significant p-values <0.05.

### 3.4 Relationships between soluble markers of HIV immune activation and immune coreceptor assessment

We used a non-parametric Spearman test to evaluate the associations between age, CD4+, CD8+ T cell counts, pVL, CD4/CD8 ratio, sCD163, sCD14 and IL-6 in 98 individuals. We found negative and positive correlations between IL-6 and CD4+ T cell counts, pVL, CD4/CD8 ratio and sCD163 (rho = -0.440, p = <0.001; rho = 0.285, p = 0.014; rho = -0.337, p = 0.003; rho = 0.370, p = 0.001; respectively). Then, with the stratified individuals categorized into only R5 viral variants and Mixed R5 and X4 viral variants, we evaluated the same variables mentioned above. We found in the R5 group that CD4+, CD8+ T cell counts and CD4/CD8 ratio were inversely correlated with IL-6 (rho=-0.437, p=<0.001; rho=-0.267, p=0.039; rho=-0.313, p=0.013; respectively). Meanwhile, both levels of sCD163 and pVL were positively correlated with IL-6, (rho =0.366, p = 0.004; rho = 0.343, p = 0.007; respectively). In the X4 group, the results showed that only sCD163 and sCD14 were inversely correlated with CD4/CD8 ratio (rho =-0.638, p = 0.046; rho = -0.632, p = 0.046; respectively).

### 3.5 Principal component analysis

**Figure 2:**
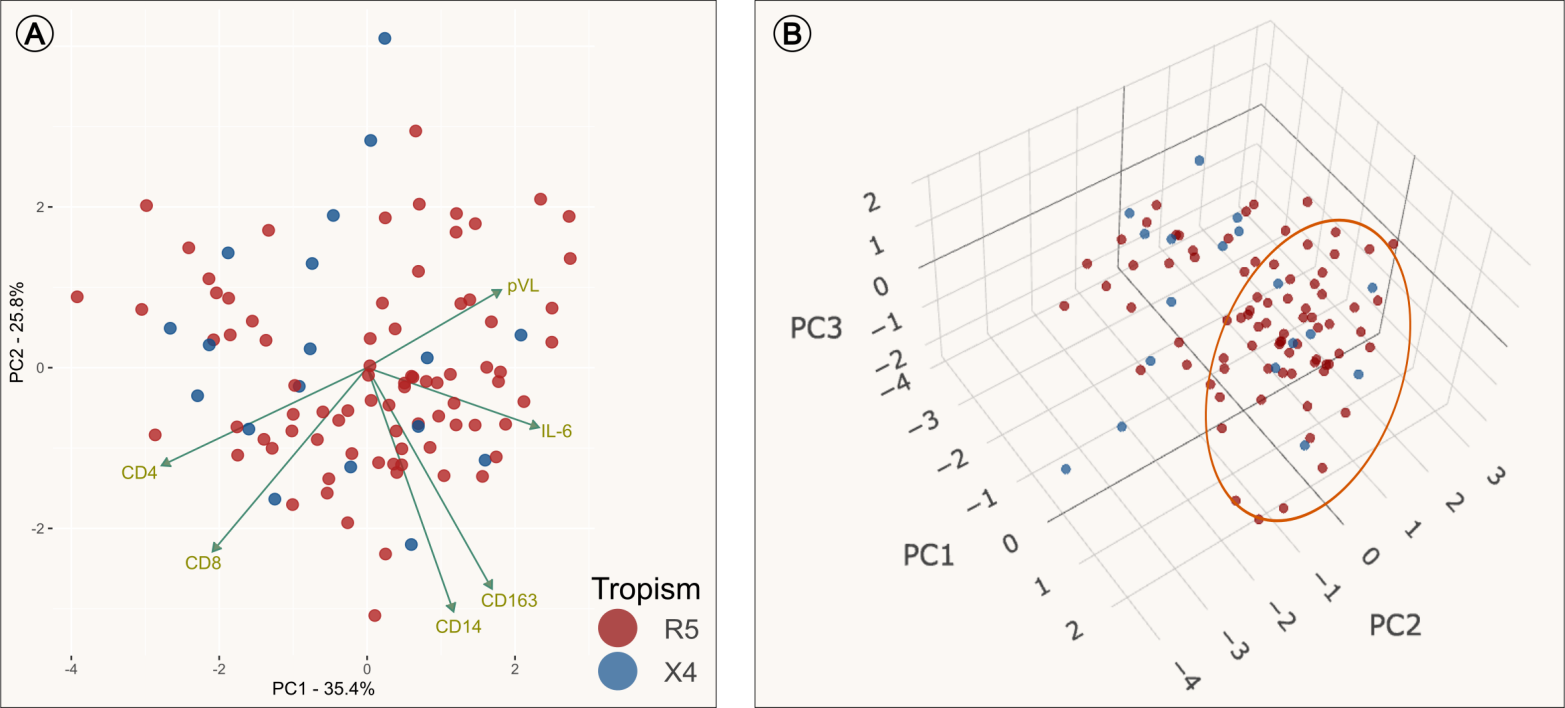
Principal Component Analysis (PCA). Dimension reduction analysis of the transform clinical and immune set of variables into a smaller one that still contains most of the information in the original set. PCAs colored by tropism assessment: individuals with only R5 viral variants (red), individuals with presence of X4 viral variants (blue) and loading values represented by green arrows. **(A)** PCA plot using data from PC1 (principal component) and PC2. It is observed that the loading value of IL-6 was inversely correlated with CD4 and CD8; and positively correlated with pVL. **(B)** PCA plot of three dimensions (PC1, PC2 and PC3). The group clustered along the left side of the PC1 are the individuals who had increased levels of IL-6 as the mayor variable that contributes to PC1 (Orange circle).

Subsequently, a PCA was conducted to examine whether PLWH who exhibited similar immunological characteristics associated with immune activation would cluster according to coreceptor assessment. The objective of this test was to identify whether the three soluble markers (sCD14, sCD163 and IL-6) and some clinical characteristics (CD4+ count, CD8+ count and pVL) could provide insight into the underlying presence of X4 viral variants (**Figure 2**). As illustrated in **Figure 2A**, the R5 and X4 groups were not well separated from each other, although the majority of PLWH were positioned along the right side of the plot, which is distinctly defined by principal component 1 (PC1). The PC1 loading values with the greatest impact on PC1 were IL-6 and CD4+ T cells count (see **Supplementary Table 2**). Also, R5 group was widely dispersed over the plot, but most PLWH were clustered on the right side and have increased levels of IL-6 and low counts of CD4+ lymphocytes (data not shown). Notably, only six X4 PLWH were clustered on the same side and also had increased levels of IL-6. To evaluate the overall significance of the PCA we used *PCAtest* (42), which assesses each PC axis and estimate the contribution of each variable to the significant axes (see **Supplementary Figure 2**). The first two PC axes were significant and accounted for 61.2% of the total variation. This evaluation reflects non-random correlations among variables.

### 3.6 IL-6 as a predictor variable for the presence of X4 viral variants

Next, we performed a multiple logistic regression analysis, in order to evaluate whether age, number of viral variants, sCD14, sCD163, IL-6, pVL, CD4+ and CD8+ count could have a potential effect on the presence of X4 viral variants in PLWH. The predictor variable, IL-6, in the logistic regression analysis was found to contribute to the adjusted model (see **Supplementary Table 3**). The unstandardized Beta (β) weight for the predictor variable (IL-6) was β = -2.79, SE = 1.02, Wald = -2.74, p = 0.006. The multivariate analysis showed a decreased probability by 94% (AOR= 0.06, 95% IC [0.006-0.34]) for the presence of X4 viral variants in PLWH for every one-unit increase in IL-6. The remaining variables were not associated with the presence of X4 viral variants.

### 3.7 Differential immune activation markers CD38+ and HLA-DR+ CD38+ in X4 vs R5 viral variants

**Figure 3:**
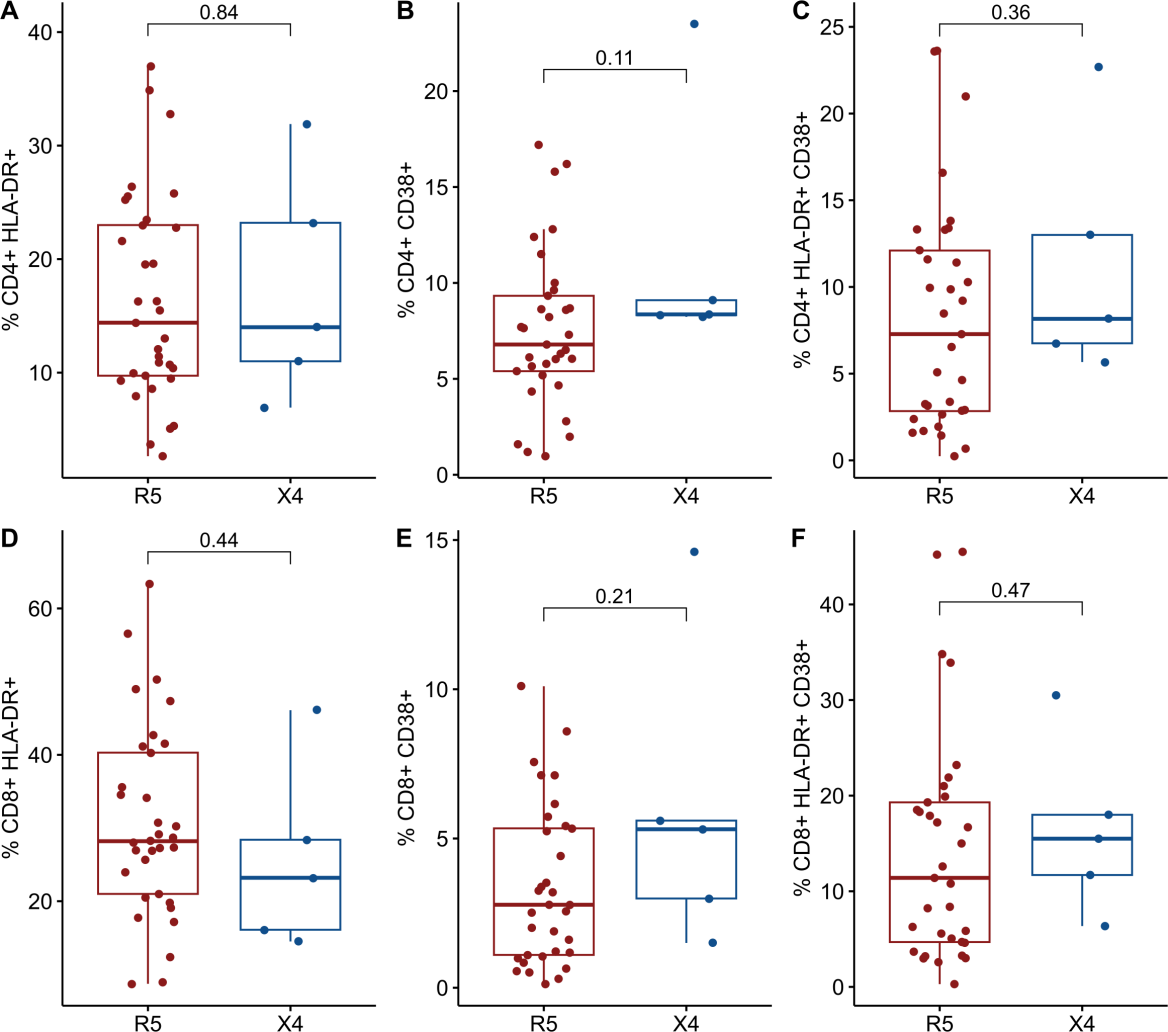
Comparisons of cellular markers of immune activation between R5 group (red) and X4 group (blue). The percentages of activated CD4+ T cells and CD8 T cells are plotted among 98 in PLWH. **(A)** %CD4+ HLA-DR+. **(B)** %CD4+ CD38+. **(C)** %CD4+ HLA-DR+ CD38+. **(D)** %CD8+ HLA-DR+. **(E)** %CD8+ CD38+. **(F)** %CD8+ HLA-DR+ CD38+. CD, cluster of differentiation; HLA-DR, Human Leukocyte Antigen – DR. Comparisons were made using Mann-Whitney U test or t-test as appropriate and using significant p-values <0.05.

**Figure 4:**
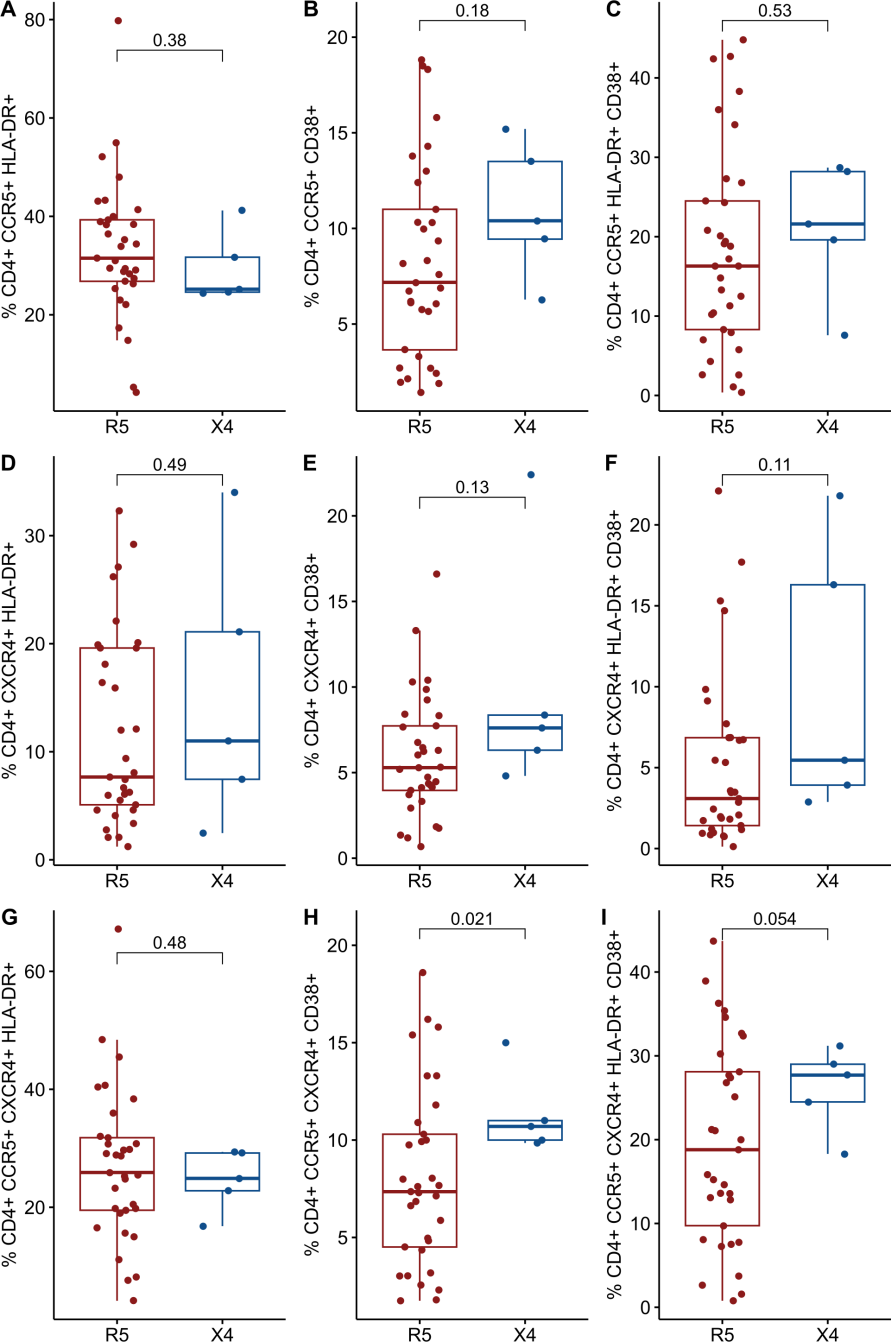
Comparisons of cellular markers of immune activation between R5 group (red) and X4 group (blue). The percentages of activated CD4+ T cells and CD8 T cells are plotted among 98 in PLWH. **(A)** %CD4+ CCR5+ HLA-DR+. **(B)** %CD4+ CCR5+ CD38+. **(C)** %CD4+ CCR5+ HLA-DR+ CD38+. **(D)** %CD4+ CXCR4+ HLA-DR+. **(E)** %CD4+ CXCR4+ CD38+. **(F)** %CD4+ CXCR4+ HLA-DR+ CD38+. **(G)** %CD4+ CCR5+ CXCR4+ HLA-DR+. **(H)** %CD4+ CCR5+ CXCR4+ CD38+. **(I)** %CD4+ CCR5+ CXCR4+ HLA-DR+ CD38+. CD, cluster of differentiation; HLA-DR, Human Leukocyte Antigen – DR; CCR5, C-C chemokine receptor 5; CXCR4, C-X-C chemokine receptor 4. Comparisons were made using Mann-Whitney U test or t-test as appropriate and using significant p-values <0.05.

The expression of cellular markers was finally measured in 38 cryopreserved PBMC from the INER group to assess levels of chronic immune activation. The IMSS group had no available cryopreserved PBMCs. The 38 PLWH were stratified into two groups in a manner consistent with that previously described. Only five PLWH were found to harbor viral variants that use CXCR4 as a coreceptor, while the remainder (n = 33) of the PLWH were found to harbor only R5 viral variants. No differences were observed between the R5 and X4 groups (**Table 4**). As illustrated in **Figure 3**, the X4 group exhibited increased levels of CD38+ and both HLA-DR+ CD38+ in CD4+ and CD8+ T cells. It is noteworthy that the frequency of CD4+ CCR5+ CXCR4+ CD38+ cells was significantly elevated in the X4 group (7.8 vs 10.7, p = 0.021) (**Figure 4**).

**Table 4:** Cellular markers of immune activation in T CD4+ and CD8+ lymphocytes.

Markers	All individuals Median (Q1-Q3) n = 39	CCR5 (FPR >3.75) Median (Q1-Q3) n = 34	CXCR4 (FPR <3.75) Median (Q1-Q3) n = 5	p value
CD4+ T Lymphocytes, coreceptor; and markers of senescence and exhaustion				
*% CD4+*	10.3 (5.722-17.25)	10.4 (7.12-19.4)	5.49 (3.67-12.1)	0.159
*% CD4+ CCR5+*	5.68 (3.283-10.975)	5.44 (3.13-11.2)	7.7 (4.82-9.84)	0.769
*% CD4+ CXCR4+*	56 (33.08-67.95)	56.4 (33.6-68.1)	39.2 (21.9-62.8)	0.472
*% CD4+ PD-1+*	39.2 (25.10-42.33)	39 (26.3-42)	58.8 (20.1-78.1)	0.328
*% CD4+ CD57+*	8.59 (2.925-11.9)	8.24 (2.1-11.2)	11.6 (6.22-20.2)	0.218
CD4+ T Lymphocytes, Markers of cellular activation				
*% CD4+ HLA-DR+*	14.2(9.78-23.15)	14.4 (9.73-23)	14 (11-23.2)	0.837
*% CD4+ CD38+*	7.67 (5.68-9.27)	6.79 (5.4-9.33)	8.36 (8.32-9.1)	0.112
*% CD4+ CD38+ HLA-DR+*	7.72 (2.97-12.78)	7.28 (2.84-12.1)	8.16 (6.75-13)	0.364
CD8+ T Lymphocytes markers of senescence and exhaustion				
*% CD8+*	78.6 (63.43-83.85)	78.6 (63.2-83.4)	84.1 (81.5-85.9)	0.142
*% CD8+ CD57+*	14.6 (8.61-21.23)	14.8 (8.44-21.3)	9.25 (9.12-17.8)	0.635
*%CD8+ PD-1+*	20.2 (14.28-24.35)	20.1 (14.1-23.6)	22.7 (21.4-24.6)	0.461
CD8+ T Lymphocytes, Markers of cellular activation				
*% CD8+ HLA-DR+*	28.1 (20.63-39.13)	28.2 (21-40.3)	23.2 (16.1-28.4)	0.441
*% CD8+ CD38+*	2.89 (1.18-5.4)	2.78 (1.1-5.34)	5.31 (2.99-5.6)	0.210
*% CD8+ CD38+ HLA-DR+*	12.15 (5.19-19.1)	11.4 (4.69-19.3)	15.5 (11.7-18)	0.475

IQR, Interquartile range; CD, cluster of differentiation; HLA-DR, Human Leukocyte Antigen – DR; CCR5, C-C chemokine receptor 5; CXCR4, C-X-C chemokine receptor 4; PD-1, Programmed cell death protein-1. Comparisons were made using Mann-Whitney U test or t-test as appropriate and using significant p-values <0.05.

### 3.8 Spearman correlation analysis of immune activation, senescence, and exhaustion markers reveals bidirectional correlations with CCR5+ and CXCR4+ coreceptor expression in CD4+ and CD8+ T cells

A non-parametric spearman test was used to explore the relationship between cellular immune activation and the expression of CCR5+ and CXCR4+ coreceptors in CD4+ T cell (**Supplementary Figure 4**). This analysis was derived from a sample of 38 PLWH from the INER cohort. We found moderate negative correlations specifically between CD4+ T cells and exhausted CD4+ PD1+ (rho = -0.530, p = 0.004) and markers of cellular activation on CD4+ T cell which express CXCR4+ coreceptor (CD4+ CXCR4+ HLA-DR+ CD38+; rho = -0.425, p = 0.033). Conversely, CD8+ T cells exhibited a positive correlation with CD4+ PD1+ (rho = 0.409, p = 0.042); and CD8+ T cells with a marker of cellular activation in CD4+ T cells (CD4+ HLA-DR+ and CD4+ CXCR4+ HLA-DR+; rho = 0.593, p = <0.001 and rho = 0.565, p = 0.001, respectively). In regard with the expression of CCR5, CXCR4 or both in CD4+ T cells, we found that CD4+ PD1+ and CD4+ CD57+ were positively correlated with CD4+ T cells that express CCR5 (rho = 0.572, p = 0.002; and rho = 0.563, p = 0.002, respectively) or both (rho = 0.402, p = 0.05; and rho = 0.445, p = 0.03, respectively). Meanwhile, CD4+ CXCR4+ was inversely correlated with both markers PD1+ and CD57+ (rho = -0.639, p = <0.001; rho = -0.567, p = 0.002, respectively). CD4+ PD1+ was positively correlated with the expression of both markers of cellular activation (CD4+ HLA-DR+ CD38+; rho = 0.448, p = 0.023), also among the CD4+ T cells expressing the CXCR4 coreceptor (CD4+ CXCR4+ HLA-DR+ CD38+; rho = 0.593, p = 0.001).

## 4 Discussion

In this cross-sectional study, we assessed coreceptor usage by using a highly sensitive assay to detect X4 viral variants and examined levels of chronic immune activation in PLWH. Although no statistically significant values were observed, our data appear to be consistent with the increased levels of CD38+ alone and HLA-DR+CD38+ in the X4 group in relation to the R5 group (see **Figures 3**, **4**), providing suggestive data of T cell activation. However, our data did not provide sufficient evidence to conclude that PLWH harboring X4 viral variants exhibit elevated levels of markers of immune activation. Conversely, evidence was provided that PLWH exclusively harboring the R5 virus exhibited increased levels of IL-6 in comparison to PLWH with a mixed R5 and X4 virus population.

The initial report, which sought to establish a relationship between the cellular markers of activation and the emergence of X4 viral variants, was conducted by Connell and colleagues (7). This study observed that PLWH with X4 viral variants exhibited elevated levels of HLA-DR+ CD38+ in CD4+ T cells. Moreover, at five years of follow-up, a positive correlation has been identified between X4-tropism and CD4+ HLA-DR+ T cells and CD4+ HLA-DR+ CD38+ T cells (7). It has been demonstrated that the expression of CD38+ alone in CD8+ T cells is strongly associated with disease progression, irrespective of the CD4+ T cell count. This provides evidence that CD38+ expression in CD8+ T cells is a useful marker of lymphocyte activation in studies such as those conducted by Giorgi JV and colleagues (22, 43, 44). Furthermore, research has been conducted on the expression of CD38+ alone, or in combination with HLA-DR+ in CD4+ T cells. This research revealed that CD38+ expression in CD4+ T cells is an effective prognostic marker of impaired immune function (19, 45–48). One hypothesis that may explain the relationship between cellular activation, particular that of CD4+ T cells, and the presence of X4 virus with the deterioration of the immune system, is the high cytotoxicity of the X4 virus in relation to the R5 virus (8, 11, 49, 50). Holm and colleagues (46) have demonstrated that the infection of primary T cell cultures with the CXCR4-tropic HIV-1 variant ELI6, induce activation of CD4+ and CD8+ T cells, which subsequently undergo apoptosis. Additionally, this activation is attributed to the presence of replicating and non-replicating viruses (46, 51). In contrast, Meditz et al. (45) report that the primary source of HIV-1 propagation in lymph nodes was the activated CD4+ T cells expressing HLA-DR+ CD38+ and high levels of CCR5 which are the main target of R5 virus. This is likely the main cause for CD4+ T cell depletion in lymphoid tissue. Furthermore, in the same study, *in vitro* models have demonstrated that most R5 and X4 viruses replicate in CD4+ T cells expressing CD38+, irrespective of which coreceptor is expressed on these cells. This suggests that the depletion of CD4+ T cells is driven by either direct cell lysis or bystander apoptosis. The precise mechanism by which coreceptor tropism contributes to this process will be discussed in more detail later. However, it is evident that activated lymphocytes are central to the virus’s propagation.

It is noteworthy that when CD4+ T cells expressing both CCR5+CXCR4+ coreceptors and CD38+ were selected, a significant difference was found between R5-tropism PLWH and X4-tropism (7.8 vs 10.7, p = 0.021, see **Figure 4**). The underlying reasons for the observed discrepancy compared to CD4+ T cells expressing only CD38+ require further discussion. One plausible hypothesis is that the small sample size of the X4 group (n=5) may have influenced the observed outcomes. The data suggest that PLWH within this group exhibited elevated markers of immune activation, although not to a significant extent, a limitation previously acknowledged by Cassadella et al. (6, 52). These authors propose that implementing NGS technology in conjunction with a more stringent FPR threshold, could refine the identification of PLWH harboring the X4 virus. Such an approach would likely reduce the number of PLWH who are stratified within the X4-group, thereby addressing the current limitations in statistical power due to sample size constraints. Additionally, it is also interesting to note that circulating central memory CD4+ T cells express both coreceptors (CCR5 and CXCR4) and exhibit an activated phenotype characterized by CD38 expression (53). These cells reside outside the GALT. Given that the cells analyzed in this study were derived from PBMC, this may explain why significant levels of cellular activation were specifically observed in these cells. However, no differences were detected between groups in terms of lymphocyte subtypes (data not shown), nor was there any evidence to suggest that the expression levels of coreceptors predispose PLWH to the presence of X4 variants. Importantly, the relationship between cellular activation and the emergence or presence of X4 variants does not necessarily imply changes in coreceptor expression levels or the co-expression of both markers associated with cellular activation (HLA-DR+ and CD38+) (10, 54).

In this study, the hypervariable V3 region of HIV-1 was analyzed using the online platform geno2pheno, which provides false-positive rate (FPR) cutoffs to estimate the probability of incorrectly classifying R5-tropic virus sequences as X4-tropic. A lower FPR indicates a reduced likelihood of misclassifying X4-tropic sequences. To characterize highly diverse viral populations, we employed the Illumina MiSeq platform, which offers significant advantages over other next generation sequencing platforms by minimizing errors associated with homopolymers, insertions, deletions, and other sequencing artifacts. Additionally, this technology allows for increased sequencing depth per sample, reduces error rates, and avoids artifacts derived from PCR-mediated recombination. These advantages enabled accurately identifying and quantifying of low-frequency X4-tropic viral subpopulations within a broader set of viral quasispecies. However, inherent issues with the technology, such as PCR resampling and PCR/sequencing errors, limit the study of these low-frequency variants. In order to address these issues, a number of studies have developed unique molecular identifiers (not applied in this study), such as Primer ID (55–57), with the aim of identifying true low-frequency variants and reducing errors in genetically diverse populations. In this study, we explore different cut-off points for the viral population, with thresholds of >2% and >5% (a more conservative approach) being examined. Our findings indicate that there are no discrepancies in the stratification of the PLWH by tropism when both cut-offs are employed (data not shown).

In this cohort, the median number of viral variants was 1836 (IQR = 937.5 - 2942.5), reflecting the high viral diversity and genetic variability of HIV-1. The prevalence of X4-tropic viruses in late HIV presenters was 18.4%, consistent with findings reported in other studies (16, 17, 39, 52, 58–60). In contrast, in PLWH with advanced disease stages, up to 50% of PLWH have been documented to harbor X4-tropic viruses. The remaining viral variants likely represent populations with reduced viral fitness, which appear and disappear continuously. However, these variants may play a relevant role in CD4+ T-cell depletion, either through direct lysis or bystander apoptosis. Furthermore, it has been observed that the gp120 can spontaneously shed from the surface of virions and infected cells, suggesting a potential role in modulating immune responses. Variable levels of soluble gp120 have been reported in PLWH (61). This soluble form of gp120 has been shown to interact with the CD4 receptor on T lymphocytes, monocytes, dendritic cells, and macrophages, inducing the secretion of proinflammatory cytokines such as IL-6, IL-10, IL-1β, interferon-alpha and gamma, and tumor necrosis factor-alpha (TNF-α) (62). These findings highlight the importance of gp120 in not only viral entry but also in the pathogenesis of HIV through the activation of inflammatory responses.

With regard to the clinical characteristics exhibited by the cohorts under investigation, significant differences were identified in CD4+ T cell count and pVL. The INER cohort was comprised of PLWH who had probably delayed even more time their diagnosis and treatment for various reasons. As tertiary-level of care hospital, most PLWH arrived with advance disease. By contrast the IMSS cohort exhibited favorable CD4+ and pVL values, which could be indicative of reduced levels of soluble markers (sCD14, sCD163 and IL-6; data not shown). Conversely, no significant differences were observed between the R5 and X4 groups with respect to CD4+, CD8+ T cell count and pVL. Connell and colleagues (7) observed a negative association of X4-tropism and CD4+ T cell count; however, our findings related to CD4+ T cell count did not show significant differences between both groups. It is acknowledged that a potential confounder in this study is the fact that over 60% of the PLWH in both groups had CD4+ T cells <200 cells/µL, a likely consequence of late diagnosis of HIV-1 in these individuals. The low CD4+ T cell count at the time of diagnosis indicates that the majority of PLWH were at a stage of advanced disease, which is also reflected by elevated levels of pVL. Despite the sustained efforts to implement strategies to initiate early treatment with antiretroviral therapy in Mexico, late diagnosis remains frequent among individuals attending health services due to fear of discrimination, stigma, and social inequalities, in particular with respect to access to health care. Consequently, the stratification of these individuals with early-stage disease was complicated. Clinical data presented in this study is consistent with the findings of previous studies (63, 64), which reported that more than 50% of individuals were diagnosed and treated in a late stage of the disease in Mexico. These individuals typically presented with a CD4+ T cell count of less than 200 cells/mm^3^ and/or a history of AIDS-defining illness.

In this study, levels of sCD163 are consistent with Connell and colleagues findings (7), who identified elevated levels of sCD163 in the R5 group, though not significant. In some studies, this soluble marker has been identified as a useful predictor of mortality and morbidity and is associated with elevated plasma viral load (31, 65, 66). Regarding sCD14, similar values were observed across groups. Given that these PLWH were diagnosed at a late stage, it is plausible to infer a process of monocyte/macrophage stimulation via LPS, which is characteristic of the chronic phase of infection. This is associated with elevated levels of IL-6, immune activation, gut dysbiosis, and microbial translocation (30, 67).

In contrast, our study found that IL-6 was significantly elevated in the R5 group. This finding appears counterintuitive, as it would be hypothetically expected that the X4 group would cause greater immune activation. To date, there are no reports linking elevated IL-6 levels to the presence of X4 viral variants in PLWH. Nonetheless, there are a few reports where IL-6 is strongly induced by gp120. Del Cornò, et al. (68) showed that the engagement of coreceptor CCR5 by a HIV-1 R5-tropic gp120 promotes STAT3 activation concentration-dependently and time-dependently leading to IL-6 secretion by immature monocyte-derived dendritic cells. STAT3 belongs to family of proteins known as signal transducers and activators of transcription (STAT), which are involved in diverse biological processes, such as cell differentiation, apoptosis and inflammatory response (69, 70). In order to corroborate the involvement of the coreceptor, the authors used a CCR5 antagonist, Tak779, which blocks the function of the coreceptor CCR5 and the signaling mediated by CCL4 (the natural ligand of CCR5). CCL4 did not induce the secretion of IL-6 by itself, which confirms that this ligand does not promote STAT3 activation in a natural manner. Moreover, Shah, et al. (71) showed that gp120 increased the expression of IL-6 at both mRNA and protein levels in astrocytes. These cells express both coreceptor CXCR4 and CCR5. Their findings indicated varying levels of IL-6 expression in response to several strains of gp120, including X4-tropic gp120IIIB which exhibited lower expression levels compared with R5-tropic gp120 CN54, gp120 CM and gp120 Bal strains. The involvement of the coreceptor in the viral entry mechanism may also initiate signaling events that can modify the cellular function or impact the post-entry stage of infection. Therefore, it can be demonstrated that CCL4 does not intervene in the secretion of IL-6, nor can it be excluded that CXCR4 does not implicate in the activation of STAT3. The hypothesis that lower levels of IL-6 in PLWH with X4 viruses could promote IL-6 secretion via STAT3 to a lesser degree is therefore proposed, as suggested by the aforementioned results.

Furthermore, principal component analysis was performed to assess interdependencies among the studied variables. This analysis revealed a significant negative correlation between CD4+ T cell counts and IL-6 levels, consistent with our findings using Spearman test described earlier. In the PCA, IL-6 emerged as a positive vector, clustering subjects harboring R5-tropic viruses on the right side of the biplot. In contrast, CD4+ T-cell counts were represented as a negative vector, grouping PLWH with X4-tropic viruses on the left side. Notably, the soluble markers sCD14 and sCD163 did not contribute substantially to the variance explained by the first principal component (PC1). However, these markers aided in distinguishing PLWH with elevated levels of soluble markers, which were positioned on the right side of the graph. It is worth noting that the majority of PLWH exhibited markedly low CD4+ T cell counts, which may account for the observed dispersion in the PCA plot and the absence of well-defined clusters separating the X4 and R5 groups. Moreover, a multiple logistic regression model was used to evaluate the predictive value of the variables. The model underscored the significance of IL-6, indicating that elevated levels of this cytokine may serve as a predictive biomarker for the presence of exclusively R5-tropic viral populations. These findings are consistent with the hypothesis that R5-tropic viruses are associated with an enhanced inflammatory response, as reflected by increased IL-6 levels, whereas X4-tropic viruses may associate with a less pronounced inflammatory state but exhibit greater cellular activation in terms of CD38+ and/or HLA-DR+ expression, leading to greater CD4+ T-cell depletion.

It is hereby proposed that the HIV-1 T/F R5-tropic viruses are the primary causative force of T cell activation and significant CD4+ T cell depletion, particularly within the gut-associated lymph tissue during the initial phase of the disease. The mucosal site represents the primary route of viral entry into the host, wherein a significant proportion of immune cells express the CCR5 coreceptor (e.g. macrophages, dendritic cells, and memory CD4+ T cells), thereby conferring a selective advantage to the R5-tropic viruses over X4-tropic viruses (3, 35). The R5 viruses compete with X4 viruses for the cellular targets that express both coreceptors, especially those activated CD4+ T cells, as these cells are metabolically more active, thus providing a highly favorable environment for replication (72). This dynamic limit the replication of the X4 viruses, consequently leading to a reduced production of the virions. In addition, the R5-tropic viruses have been distinguished by their increased viral burst, defined as the number of virions released per infected cell (10). Therefore, it is rarely observed that the X4 viruses predominate during the early stages of the disease. We propose that the relationship between coreceptor switching and immune activation is bidirectional; in one direction, immune activation facilitates the change of coreceptor use by creating an environment more suitable for the selection of X4 viruses. In the absence of antiretroviral therapy, the infection process gives rise to the emergence of competent viruses that exhibit diverse phenotypes. This phenomenon is a direct consequence of the host environment undergoing constant alterations in response to the dynamic interplay between the virus and its host. Consequently, this process generates a series of selective pressures that favor proliferation of variants better adapted to the prevailing environmental conditions (3, 51, 72). In the opposite direction, at the late stage of infection, the X4 virus has been observed to diminish immune system function by means of the activation of additional CD4+ T cells and the more efficient destruction of immune cells, thus perpetuating the inflammatory cycle and immune damage (8, 46, 49–51). It is imperative to better understand the interconnected processes of chronic immune activation and coreceptor switching in HIV infection, as these may associate to the progression of the disease. Elucidation of these mechanisms is essential for the formulation of therapeutic strategies that modulate immune activation and prevent viral tropism switching. The objective of such strategies is to preserve immune function and enhance clinical outcomes in individuals living with HIV.

During the course of this study, we encountered several limitations. Firstly, we were limited in reporting causality due to the cross-sectional nature of this study. Secondly, as previously mentioned, the statistical power of our sample size in the X4 group was limited. Thirdly, there was an absence of available PBMCs in the IMSS cohort. These limitations might account for the lack of statistical differences when comparing markers of immune activation between the R5 and X4 groups. Lastly, the majority of the PLWH recruited in both cohorts showed reduced CD4+ T cell counts, suggesting prolonged viral exposure, which could result in a sustained immune activation with increments in the levels of several biomarkers in individuals with greater time exposure. The study could not determine whether participants had acute or early HIV infection, thus Fiebig staging could not be performed. Lastly, unmeasured confounders may have influenced levels of cytokine and immune activation, in particular coinfections with cytomegalovirus, hepatitis C and B, and opportunistic infections, and smoking. These confounders were not recorded for most of PLWH in our study, limiting our ability to include them in our analyses. Contrastingly, the study’s strengths are supported by the use of advanced technological instruments, such as the employment of flow cytometry for the quantification of immune activation markers and the integration of paired-end sequencing data with the geno2pheno algorithm for the identification of individuals harboring X4 variants. Furthermore, the participants in this study represent a diverse sample of the PLWH residing in the metropolitan area of Mexico City.

## 5 Conclusion

This study explored coreceptor usage and chronic immune activation in late HIV-1 presenters, revealing trends of elevated CD38+ and HLA-DR+CD38+ in CD4+ or CD8+ or both expression in the X4 group and significantly higher IL-6 levels in the R5 group. While no statistically significant differences were observed in the cellular markers of inflammation, the data is consistent with prior research (7). The findings suggest that R5-tropic viruses drive inflammatory responses in early stages, while X4-tropic viruses may enhance CD4+ T cell depletion through immune activation. These insights highlight the complex interplay between viral tropism and immune activation. Future studies with larger sample sizes and longitudinal designs are needed to confirm these findings and explore the causal relationships between viral tropism, immune activation and disease progression.

## Data Availability

The datasets presented in this study can be found in online repositories. The names of the repository/repositories and accession number(s) can be found below: https://www.ncbi.nlm.nih.gov/, PRJNA1261961.
