# Supplementary material for: Patterns of inflammation and immune activation by coreceptor use in people living with HIV-1
Source: Front Immunol. 2025 Jul 10;16:1632287. doi: 10.3389/fimmu.2025.1632287 (PMC12286835; doi:10.3389/fimmu.2025.1632287)
Supplement: Supplementary file 1 [file DataSheet1.docx]

Supplementary Material


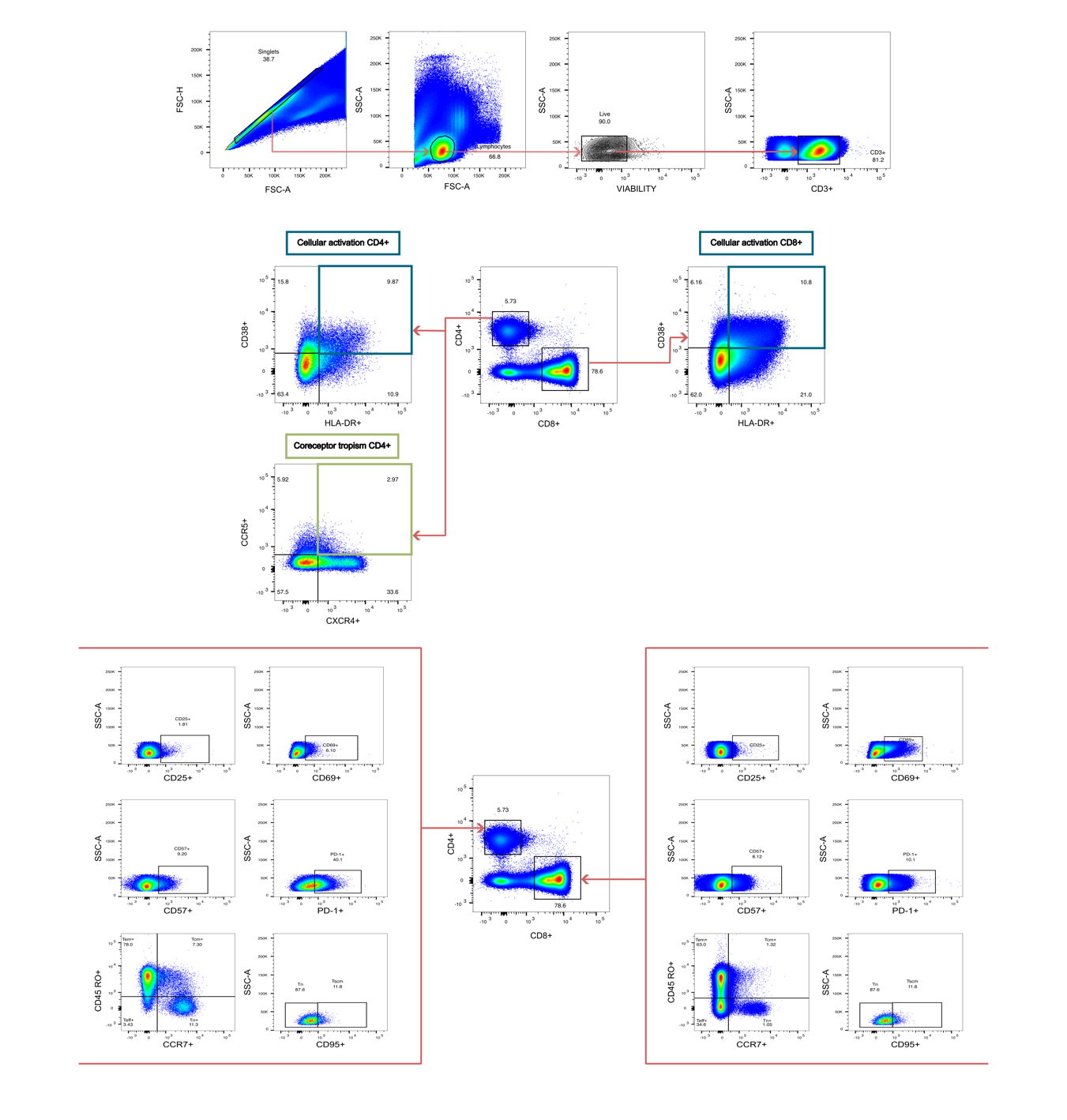


Supplementary figure 1. Gating strategy for the lymphocyte analysis. Monoclonal antibodies, including CD3, CD4, CD8, CD69, CD25, HLA-DR, CD38, CD45RO, CD197 (CCR7), PD-1, CD57, CCR5 (CD195), CXCR4 (CD184) and a Live/Dead stain kit were used in these cells. FMO (fluorescence minus one) were used to accurately set the gates for positive populations. Flow cytometry acquisition was performed on a BD LSRFortessa (BD Biosciences, San Jose, CA, USA) within 24 h of staining. A minimum of 3 million events were acquired per sample. Quality controls were performed using BD Cytometer Setup and Tracking Beads and Rainbow Beads (BD Biosciences). A compensation matrix was calculated and applied using BD Comp Beads (BD Biosciences). Data was analyzed using FlowJo™ v10.9 (BD, Ashland, Oregon, Unites States). Raw ﬂow cytometry standard (FCS) ﬁles were initially quality controlled using FlowAI v2.3.1 with default parameters. Lymphocytes were identified based on FSC (forward scatter) and SSC (side scatter) signals from peripheral blood mononuclear cells (PBMC). Initially, PBMC were gated for singlets, followed by lymphocytes, viability cells, and finally, CD3+ expressing cells. From these cells CD4+ and CD8+ were gated. The remaining cellular markers were gated from CD4+ and CD8+ cells. CD: cluster of differentiation, HLA-DR: Human Leukocyte Antigen – DR, CCR5: C-C chemokine receptor 5, CXCR4: C-X-C chemokine receptor 4, PD-1: Programmed cell death protein-1.

Supplementary Table 1. Analysis of sequencing data.

| Number of reads and viral variants | **Individuals with NGS**  **Tropism results (n=98)** | **CCR5 (FPR>3.75) (n=80)** | **CXCR4 (FPR<3.75) (n=18)** | **p value** |
| --- | --- | --- | --- | --- |
| Reads median (IQR) | 105973.5  (60190-182797.75) | 91203.5  (57245.75 - 172615.8) | 150212  (73263.5 - 196082.8) | 0.159 |
| No. of viral variants median (IQR) | 1836 (937.5 - 2942.5) | 1577 (910.25 - 2657.25) | 2870 (1132.75 - 3971) | 0.022 |

NGS: next generation sequencing, FPR: false positive rate, CCR5: C-C chemokine receptor 5, CXCR4: C-X-C chemokine receptor 4, IQR: Interquartile range.

Comparisons were made using Mann-Whitney U test or t-test as appropriate and using significant p-values <0.05.

Supplementary table 2. Contribution of variables to principal components

| Variable | **PC1** | **PC2** | **PC3** | PC4 | PC5 | PC6 |
| --- | --- | --- | --- | --- | --- | --- |
| CD4 | **26.09** | 7.18 | 2.20 | 14.43 | 9.24 | 40.87 |
| CD8 | 12.50 | **25.98** | **20.52** | 1.49 | 16.30 | 23.20 |
| pVL (Log) | 9.20 | 7.60 | **76.83** | 1.82 | 1.27 | 3.29 |
| CD163 | 16.33 | **27.44** | 0.01 | 0.95 | 47.22 | 8.05 |
| CD14 | 11.86 | **31.79** | 0.38 | 18.72 | 13.64 | 23.61 |
| IL-6 | **24.02** | 0.01 | 0.07 | 62.59 | 12.32 | 0.98 |

pVL: plasma viral load, CD: cluster of differentiation, IL: Interleukin, sCD soluble cluster of differentiation, PC: principal component


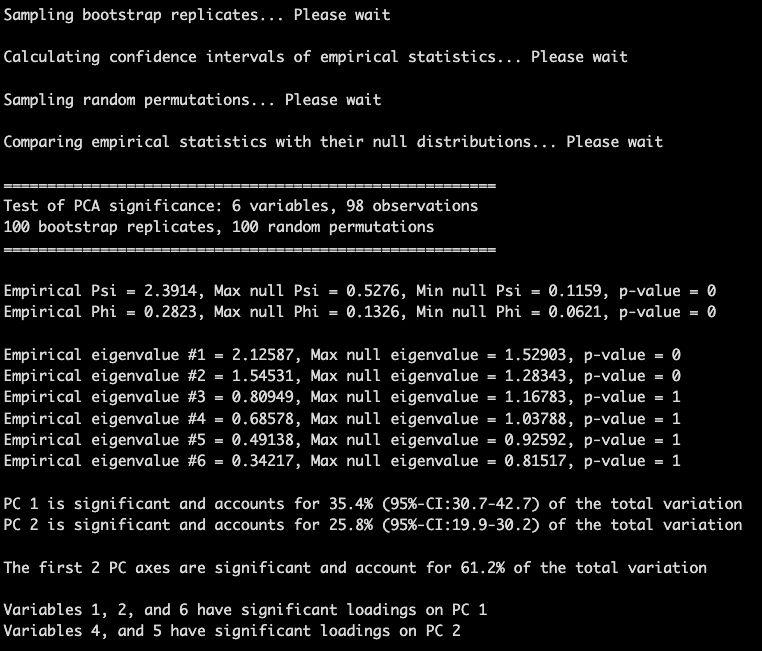


Supplementary figure 2. Results of the test of PCA significance with the R package PCAtest (ver. 0.0.1).

Supplementary table 3. Results of multivariate logistic regression model to predict the presence of X4 viral variants in PLWH.

| **Predictor** | **Estimate** | **Std. Error** | **Z value** | ***p*** | **AOR** | **95% CI** | |
| --- | --- | --- | --- | --- | --- | --- | --- |
| Age | -0.03 | 0.04 | -0.636 | 0.525 | 0.97 | 0.885 | 1.050 |
| CD4 | -0.01 | 0.00 | -2.405 | **0.016** | 0.99 | 0.979 | 0.997 |
| CD8 | 0.00 | 0.00 | 0.939 | 0.348 | 1.00 | 0.999 | 1.002 |
| pVL | -0.96 | 0.61 | -1.567 | 0.117 | 0.38 | 0.105 | 1.224 |
| N. Variants | 0.00 | 0.00 | 2.771 | **0.006** | 1.00 | 1.000 | 1.002 |
| CD163 | -0.17 | 1.71 | -0.102 | 0.918 | 0.84 | 0.025 | 25.675 |
| CD14 | -0.81 | 1.78 | -0.455 | 0.649 | 0.45 | 0.013 | 16.626 |
| IL-6 | -2.79 | 1.02 | -2.743 | **0.006** | 0.06 | 0.006 | 0.340 |
| **Null deviance:** 76.794 on 78 degrees of freedom | | | | |  |  |  |
| **Residual deviance:** 47.17 on 70 degrees of freedom | | | | |  |  |  |
| **AIC:** 65.17 | | | | **BIC:** 86.5 | | | |
| **Maximum Likelihood:** residual difference: 29.6, df: 8, p = <0.001 | | | | | | | |

pVL: plasma viral load, CD: cluster of differentiation, IL: Interleukin, sCD soluble cluster of differentiation, AOR: Adjusted Odd Ratio, CI: Confidence intervals, df: Degrees of freedom, AIC: Akaike information criterion, BIC: Bayesian information criterion, N. variants: Number of viral variants (quasispecies). Significant p-values <0.05.


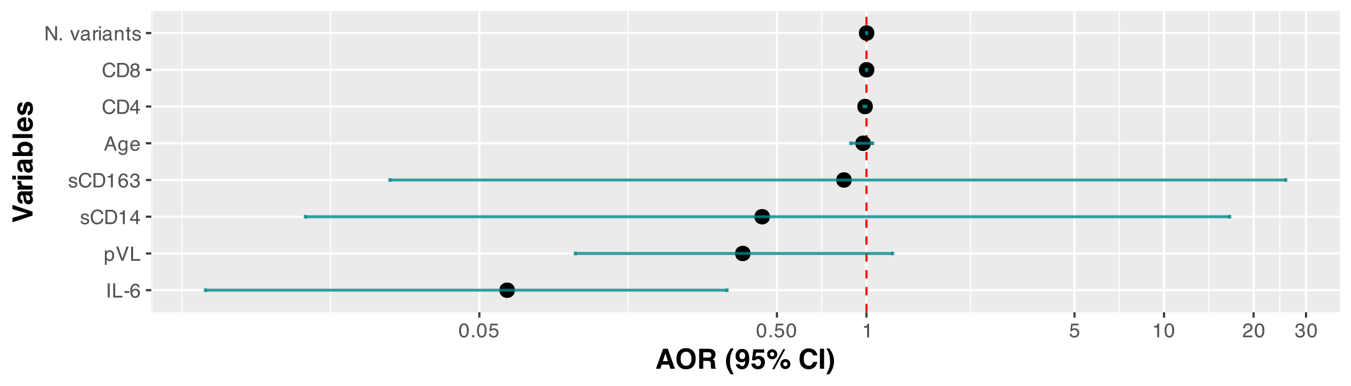


Supplemental Figure 3. Forest plot output of the multivariate logistic regression model evaluating the odds for the presence of X4 viral variants in PLWH. The predictor variables showing the adjusted odds ratio (AOR) and the confidence intervals (CI) are shown in the figure. pVL: plasma viral load, CD: cluster of differentiation, IL: Interleukin, sCD soluble cluster of differentiation, N. variants: Number of viral variants (quasispecies).


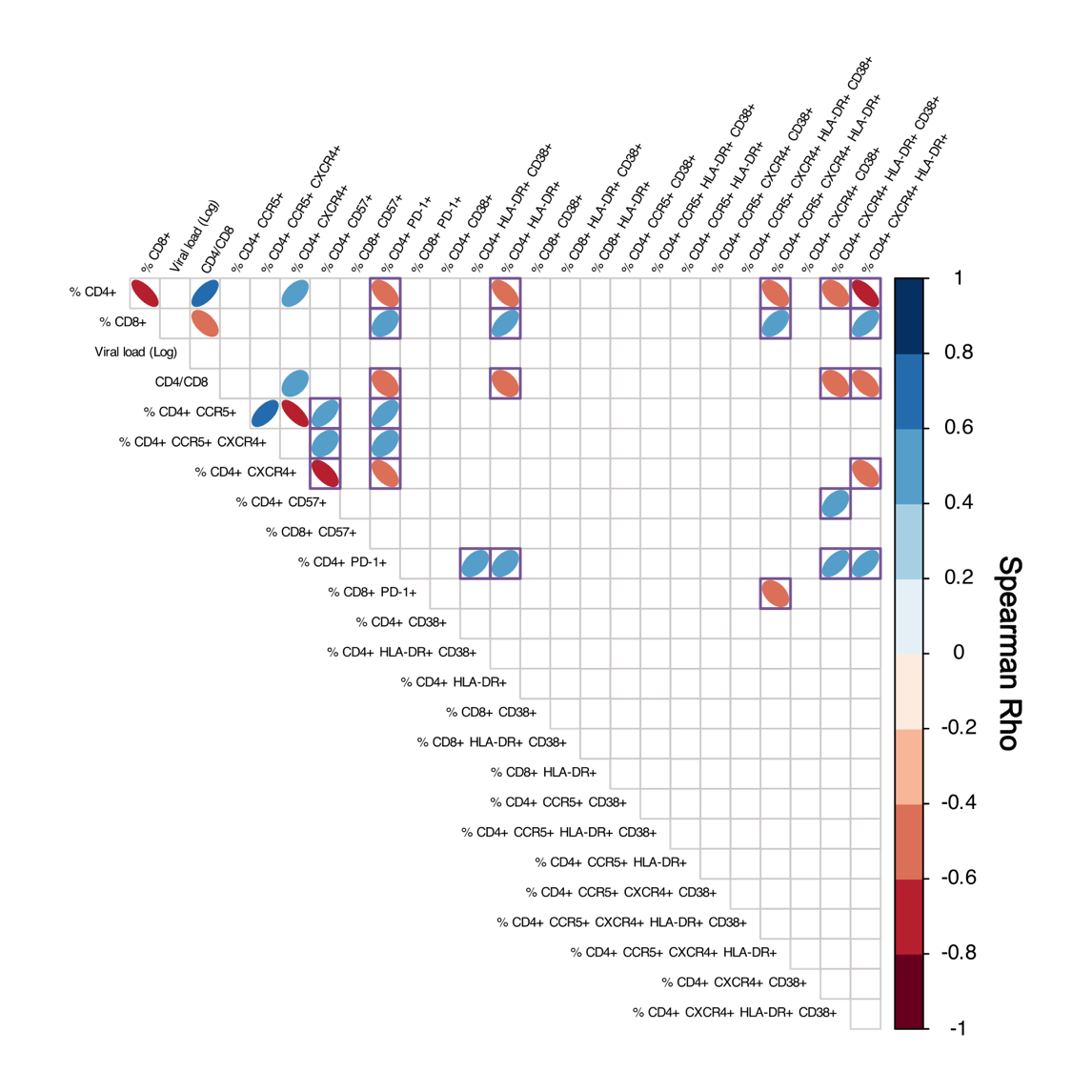


Supplementary figure 4. Graphical display of the correlation matrix illustrates the relationship between the clinical characteristics and the markers of HIV progression and immune activation. The Spearman test was used for the correlation matrix, and the Benjamini-Hochberg or false discovery rate (FDR) method was performed in order to adjust the p-values. Only correlations with significant p-values <0.05 were considered. CD: cluster of differentiation, HLA-DR: Human Leukocyte Antigen– DR, CCR5: C-C chemokine receptor 5, CXCR4: C-X-C chemokine receptor 4, PD-1: Programmed cell death protein-1.
